# Evaluation of candidate reference genes for gene expression research in *Vespula vulgaris*

**DOI:** 10.3389/finsc.2025.1495626

**Published:** 2025-02-13

**Authors:** Gemma M. McLaughlin

**Affiliations:** Independent Researcher, Christchurch, New Zealand

**Keywords:** *Vespula vulgaris*, reference gene, RT-qPCR, gene expression, invasive wasp

## Abstract

**Introduction:**

*Vespula vulgaris* is an invasive wasp that causes considerable detriment to native birds and invertebrates in New Zealand. Reducing at least 80% of invasive wasp densities is necessary to manage the problems this species presents to its invaded range. To explore the function of target genes for the genetic management of *V. vulgaris*, screening of appropriate reference genes is crucial for conducting the reverse transcriptase-quantitative real-time PCR (RT-qPCR). The selection of appropriate reference genes is an important but often overlooked consideration when delving into RNA research. Many studies rely on one of two tried and trusted reference genes widely used in the literature, which may not be suitable for the normalization of data under particular variables.

**Methods:**

Here, I selected six reference genes of *V. vulgaris* and evaluated their stability across two conditions: developmental stage and sex by using five different tools for analysis: the *ΔCt* method, *geNorm*, *NormFinder*, *BestKeeper*, and *RefFinder*.

**Results:**

Differing appropriate reference genes for different research foci: *TBP*, *EF1A*, *RPL18X3*, and *CAPZB* for developmental stage treatment, and *KTB*, *EF1A*, and *CAPZB* amongst the sexes.

**Discussion:**

My study further emphasizes that there is no “one size fits all” reference gene, and advocates for analysis of reference gene suitability when conducting gene quantification experiments.

## 1 Introduction

*Vespula vulgaris* is an invasive eusocial insect affecting suburban and beech forest areas throughout New Zealand (1). *Vespula* wasps exist at the highest global densities in New Zealand (2), to the detriment of the native avifauna and invertebrates, especially in beech forests (3–5). Extensive preliminary research is needed to lay a foundation for genetic research on *Vespula* control. The recent genome assembly and annotation of three *Vespula* genomes (*V. vulgaris*, *V. gemanica*, and *V. pensylvanica*; 6) has been a great advancement towards this, as it provides the actual sequence of genes of the organism of interest instead of relying on orthologs from closely related species from genetic databases such as NCBI. RNA interference (RNAi) approaches could be employed to silence genes pivotal to *Vespula* development. Identifying the desired target genes for silencing, then analysis of their abundance post-transcription will determine the adequacy of RNAi to control this pest species. Functional gene studies can identify the target genes ideal for silencing, and their transcript abundance can be demonstrated using quantitative real-time PCR (RT-qPCR).

RT-qPCR is an excellent method for accurately quantifying gene expression in various tissues. It evaluates the expression patterns of genes under a range of experimental conditions (7, 8) and is frequently used for interpretations of relative gene expression, providing exceptionally accurate and reproducible quantitation of gene copies with high throughput (9).

RT-qPCR is easy to use, fast, avoids the use of radioactivity, and requires a minimal amount of RNA. However, some issues reside in the use of RT-qPCR which include the variability of RNA and varying reverse transcription and PCR efficiencies (10). Unlike DNA in the genome which remains context-independent (i.e., normally cells contain the same DNA sequence), RNA in the transcriptome is context-dependent (i.e., the mRNA component level varies with pathology, physiology or development; 10), which can make it difficult to achieve an accurate result.

Ideally, such sample-to-sample variation could be normalized by measuring the levels of a single universal cellular RNA, which is present at all stages of development at constant levels, irrespective of experimental treatment or tissue used (11). These so-called reference genes are widely employed as an internal RNA reference for Northern blotting, RNase protection, and RT-qPCR practices. However, in many studies, only a single, usually popular, reference gene is used, usually without any investigation/justification for the choice of the reference gene or why they are only using one. Historically, a single reference gene has been used for RNA transcription analyses. Suzuki, Higgins and Crawford (10) found that over 90% of RNA transcription analyses published in high-impact journals in 1999 used only one reference gene. One gene in particular – glyceraldehyde-3-phosphate dehydrogenase (GAPDH) – was commonly used as the sole reference gene for RNA analysis involving human tissue biopsies, despite a lack of research supporting the suitability of this gene. GAPDH was not investigated as a suitable candidate until 2002, when GAPDH mRNA levels were found to be higher in some cancers but lower or unaltered in others in comparison to normal tissue (12) and, therefore, an unsuitable reference gene for RNA analysis. β-actin is also a frequently used control for RNA, however β-actin mRNA levels have been found to increase significantly in the adrenal glands of hypophysectomized rats exposed to adrenocorticotropin (13), increase in rat myocardium after abdominal aortic banding (14), and increases in the liver of rats with vitamin B6 deficiency (15), thus rendering it as an unsuitable reference gene when conducting such research, especially as the sole reference gene. Other papers also advocate for using more than one reference gene (see 10, 16).

Clearly, it is crucial to employ the appropriate reference genes for accurate gene expression analyses. As no experiments concerning gene expression in *V. vulgaris* currently exist, validation of suitable reference genes is necessary for accurate genetic analysis. This paper aimed to identify the most appropriate reference genes for genetic analysis in *V. vulgaris* via selection and validation of a suite of reference genes under the treatment conditions of sexes (male and female) and developmental stages (larvae, pupae, and adults). I selected 10 different reference genes as potential reference genes: dimethyladenosine transferase (*DIMT*), F-actin-capping protein subunit beta (*CAPZB*), 60s ribosomal protein (*60s RP*), peptidylprolyl isomerase domain and WD repeat-containing protein (*PPWD*), king tubby (*KTUB*), ribosomal protein L18 isoform X3 (*RPL18X3*), 18s ribosomal RNA (*18s rRNA*), adenosine kinase 1 (*AK1*), elongation factor 1-alpha-like (*EF1A*), and TATA binding (*TBP*). These genes were chosen as they have been used in previous reference gene studies in Hymenoptera (17, 18), and all are employed in the metabolic and physiological activities of cells (19, 20). The stability of these potential reference genes was initially analyzed using an RT-qPCR and evaluating standard curves. The top six genes were taken and underwent RT-qPCRs, and expression stabilities were analyzed using four different modes of software: *BestKeeper* (21), *geNorm* (16), *NormFinder* (22), and the *ΔCt* method (23). A final ranking from the cumulative data sets was also conferred via *RefFinder* (24). Based on my findings, the reference genes were ranked from most to least stable, and recommendations were made for RT-qPCR experiments in *V. vulgaris*. Finally, the DNA-dependent RNA polymerase II subunit protein RPB7 gene (*RPB7*) was used as the target gene to verify my findings. To my knowledge, this study is the first to identify stable RT-qPCR reference genes for *V. vulgaris*, which can aid in accurate gene expression analysis for *V. vulgaris* for further analysis of targeted genes for RNAi.

## 2 Methods

### 2.1 Sample collection

*V. vulgaris* nests were collected from around the Dunedin/Mosgiel region after advertising free excavation services on social media in collaboration with Nichol’s Garden Centre. Excavated nests were stored in plastic buckets in a 4°C cold room overnight to induce stasis and inhibit function. Individual adults, pupae, larvae, and adults of both sexes were then selected and stored at −80°C for RNA extraction.

Developmental stages included in this study were third-instar larvae, pupae, and three-day-old adults. Developmental stages were identified by specific features: a grub-like appearance for larvae and visualised as the middle size of the larvae present; pupae were selected from capped cells and had clear eye definition; and adults were selected upon emergence from their cells and monitored for 72 hours before freezer storage.

For the sexes, adult workers and adult drones were used. Gynes/queens were excluded from this treatment as they are genetically identical to workers and were not as accessible as workers (e.g., 15 nests with queens would have had to be excavated, as opposed to a single nest for sufficient workers).

### 2.2 Sample preparation

The stability of different reference genes in *V. vulgaris* was assessed across (i) developmental stages and (ii) sexes. Wasps used for life stages were selected the day after nest collection. Larvae and pupae were collected from nest combs, and adults were collected after emerging from their silk caps and stored in foraging boxes for three days then placed in 1.5 mL Eppendorf tubes and stored at −80°C until RNA extraction. Three samples were used for each life stage/experiment, with each experiment conducted in triplicate. Under the sexes condition, three sets of five adult workers and drones were selected separately.

### 2.3 RNA extraction

The Qiagen RNeasy Mini Kit was used for RNA extraction. Whole samples were removed from -80°C storage, transferred to a 1.5 ml Eppendorf tube, and placed on dry ice. 300 µl of Trizol was added, and the sample was homogenized using a 1000 µl pipette tip for a minimum of three minutes. More Trizol was added to make up the final volume of 1 ml, along with 200 µl of chloroform. The tube was shaken vigorously by hand for 30 seconds and then incubated at room temperature for three minutes. Samples were centrifuged at maximum speed (10,000 xg) for 15 minutes. 450 µl of the upper aqueous upper phase was transferred to a new sterile 1.5 ml Eppendorf tube with the rest discarded. 1x volume of 100%, RNA-free ethanol was added and mixed by pipetting. Samples were transferred to a RNeasy Mini spin column placed in a 2 ml collection tube, and centrifuged at 12,000 xg for one minute. The flow-through was discarded, and samples were loaded with 350 µl of buffer RW1 and centrifuged at 13,000 xg for one minute. 10 µl DNase 1 stock solution was added to 70 µl of RDD buffer, mixed by gently inverting the tube, and centrifuged briefly. This was transferred directly to the RNeasy column membrane, and incubated at room temperature for 15 minutes. The column was washed with 350 µl RW1 buffer and centrifuged for one minute at 13,000 xg with flow-through discarded. 500 µl buffer RPE was added and centrifuged as above. An additional 500 µl buffer RPE was added and centrifuged for two minutes at 8,000 xg with flow-through discarded. A “dry” centrifuge run at full spin followed to dry out the membrane. The column was transferred to a new sterile Eppendorf, and 40 µl RNase-free water was added directly to the spin column membrane. This was then incubated at room temperature for one minute and centrifuged for one minute at 8,000 xg. This was repeated once, with 5 µl aliquoted for quality assessment, while the remainder of the final samples were stored at −80°C. The 5µl aliquots were assessed for RNA purity, with 1.5 µl used for concentration quantification by measuring the absorbance at a wavelength of 206 nm with a spectrophotometer (NanoDrop 2000C, Thermo Fisher Scientific, USA), with the remainder run on a 1% agarose gel electrophoresis with a 1 Kb+ ladder (Invitrogen) and 2 µl of gel pilot loading dye (Qiagen) to check for clean bands and therefore a lack of degradation.

### 2.4 RNA purification

Samples with a 260/230 ratio of <1.5 underwent a clean-up to improve purity. A 1/10 sample volume of NaAc and 2.5x volume of 100% ethanol was added to each sample and stored at −80°C overnight. Samples were spun at 12,000 g for 30 minutes at 4°C, and the supernatant was removed. 200µl of 70% ethanol was added and samples were spun at 12,000 g for 10 minutes at 4°C. The supernatant was removed, and samples were air-dried for 5–10 minutes. Samples were resuspended in 20 µl RNase-free water and rerun in the Nanodrop to check for altered purity and concentration.

### 2.5 Verification of candidate reference genes

Candidate reference genes were first selected from *Polistes* genomes available on NCBI. Genes were identified in the *V. vulgaris* genome (Harrop et al., 2020) with diamond v0.9.24.125 using the command “diamond blastp –query proteins_of_interest.faa –db Vespula_vulgaris”. Proteins were matched to *Polistes* genome protein models and hits were further classified using visual alignment. Samtools faidx version 1.13-5-gb188dd8 was used to extract the predicted genes from the *V. vulgaris* genome where the proteins matched. Gene-specific primers were then designed using Primer Wizard (Benchling) and used to clone the open reading frame (ORF) of each reference gene. Primer sequences used for this study are shown in **Table 1**.

**Table 1:** Primers used for studying reference gene expression in *V. vulgaris* by RT-qPCR.

Gene	Primer sequence (5’–3’)	Primer efficiency (%E)	Regression coefficient (R^2^)	Tm (℃)
*DIMT*	F: TGGTGGTGGTGGATTTCTAGGT R: TGTCGCCTTAGCATCAACACGCA	268.42	0.92	61.4 58
*CAPZB*	F: ACATTGGCAGAATGGTAGAGGA R: GCTGCTTGTTGCCTTTGGTCTGC	94.42	0.98	61.7 55.9
*RP60s*	F: TGGTCCTTAGTGGTCGGTATGCTG R: ACCTGCAACCATAGCATGTCCA	263.74	0.8	62 59
*PPWD*	F: TGCAGTGCTAGTTCCTTGAGCA R: GCTGGGGATGCAATTGCTGTGG	156.2	0.96	58.8 61.6
*KTUB*	F: CCTCTTGCGTCCAGCCAGCAAA R: TGGACCGTGGTCTCTACCCTACT	101	1	62.5 60.8
*RPL18X3*	F: TCGACGCAATGCACGTGAAGCT R: GCTACGCCTACGGCCTCTAGCT	96.56	0.99	61.9 62.8
*18s rRNA*	F: TGGCGGTATGGTCGTCGATTTTCC R: CGCAGCTGGTCCTCCTGTCATT	126.27	0.99	61.2 61.7
*AK1*	F: TGGAATGGCTAGATGCGGCCCT R: CTAGCGACGGTGTGTAGCACGC	132.8	0.98	63.2 62.3
*EF1A*	F: CTCGGCGGCCTTGGTGACTTTT R: TTCCTCCGCTTGGACGTTTCGC	96.9	0.99	62.5 62.3
*TBP*	F: TGCACTGCATGAGCCGCTGAAA R: AGAGCAGCAGCCCAGCAACTTG	81.41	0.99	62.2 62.6

Reverse transcription of samples into synthesized cDNA was conducted using the following reagents: 4 µl 5x VILO™ Reaction Mix, 2 µl 10x SuperScript^®^ Enzyme Mix, 1 µg RNA, and nuclease-free water to a total volume of 20 µl. Samples were then incubated at 25°C for 10 minutes, 42°C for 60 minutes, and terminated at 85°C for 5 minutes. Samples were stored at −20°C until ready for further testing. Primers were checked for specificity via a PCR. Two controls were used: a water-only control and 100 ng of genomic DNA from a gyne sample to verify that amplification from genomic DNA results in a bigger fragment. Reagents consisted of 5 µl NEBNext^®^ Ultra™ II Q5^®^ Master Mix (Ipswich, MA), 1 µl of each of the forward and reverse primers (10 µM), 1 µl of cDNA or 3 µl of gDNA, and nuclease-free water to a total of 10 µl. Conditions of the PCR amplification comprised of 3 min at 94°C, 35 cycles of 94°C for 10 s, 58°C for 30 s, 72°C for 20 s, and a final 7 min at 72°C. Samples were visualised on a 1% agarose gel electrophoresis with a 1 Kb+ ladder (Invitrogen) and 2 µl of gel pilot loading dye (Qiagen).

To measure the efficiency of the reference genes I performed a PCR on a Light Cycler 480 thermocycler (LC480-II; Roche Diagnostics, Rotkreuz, Switzerland). An RNA sample underwent three reverse transcription reactions at 20 µl each to a pooled total volume of 60 µl. This was diluted with 240 µl of nuclease-free water to act as the undiluted initial sample. A serial dilution of cDNA (1:1, 1:10, 1:100, 1:1,000 and 1:10,000) was made with 5 µl of dilutions for each well and one water control. The qPCR reactions comprised of a master mix with 2x SYBR green mix (10 µl), 10 pmol/µl each forward and reverse primer (0.6 µl), and 3.8 µl RNase-free water to a final 15 µl volume. Each dilution was done in triplicate for each gene. he reaction cycle was as follows: 3 min at 94 °C, followed by 35 cycles of 30 s at 94°C, 30 s at 58°C, and 20 s at 72°C, and a final 7 min at 72°C. Each plate included a water-negative control for each primer pair. Analysis of the amplification plot and melting curve followed each reaction.

### 2.6 Validation and analyses of the stability of the candidate reference genes

All 10 primers underwent an initial RT-qPCR to test their amplification potential. This list was reduced to the six most effective primers, as four failed to produce stable curves across replicates.

### 2.7 Data analysis

Six reference genes with the most stable standard curves were selected for further analyses. The relative expression profiles of these genes were determined at three different developmental stages (larvae, pupae, and adults) and sexes (workers and drones). The stability of these six genes was assessed using Microsoft Excel-based software tools: *BestKeeper, geNorm, NormFinder*, and the *ΔCt* method. *BestKeeper* uses geometric means of Ct values to express the stability of reference genes. A standard deviation (SD) and stability value are used (SV); the lower SV represents more stable genes (21). *geNorm* automatically calculates the stability measure M by calculating the average pairwise variation of the reference gene against all other genes used in the analysis (16). *NormFinder* implements an ANOVA-derived model that calculates intra- and inter-group variation and then ranks the reference genes by an SV (22). The *ΔCt* method, where “pairs of genes” are compared and a rank order is determined based on mean delta Ct scores. In these analyses, genes with the lowest values are ranked as the most stably expressed for that given experimental condition. And finally, a comprehensive ranking of the stability of all candidate reference genes established from these four different statistical algorithms is employed using a Web-based analysis tool, *RefFinder* (https://www.heartcure.com.au/reffinder/; 22).

#### 2.7.1 Validation of selected reference genes

To confirm the reliability of the reference genes, the relative expression profiles of *RPB7* were determined at different development stages (larva, pupa and adult). These were normalised with the two most stable reference genes (*TBP* and *EF1A*) and the least stable reference gene (*KTUB*). Relative quantification of the target gene was calculated via the 2-ΔΔCt method (25).

## 3 Results

### 3.1 PCR amplification of candidate reference genes

**Figure 1:**
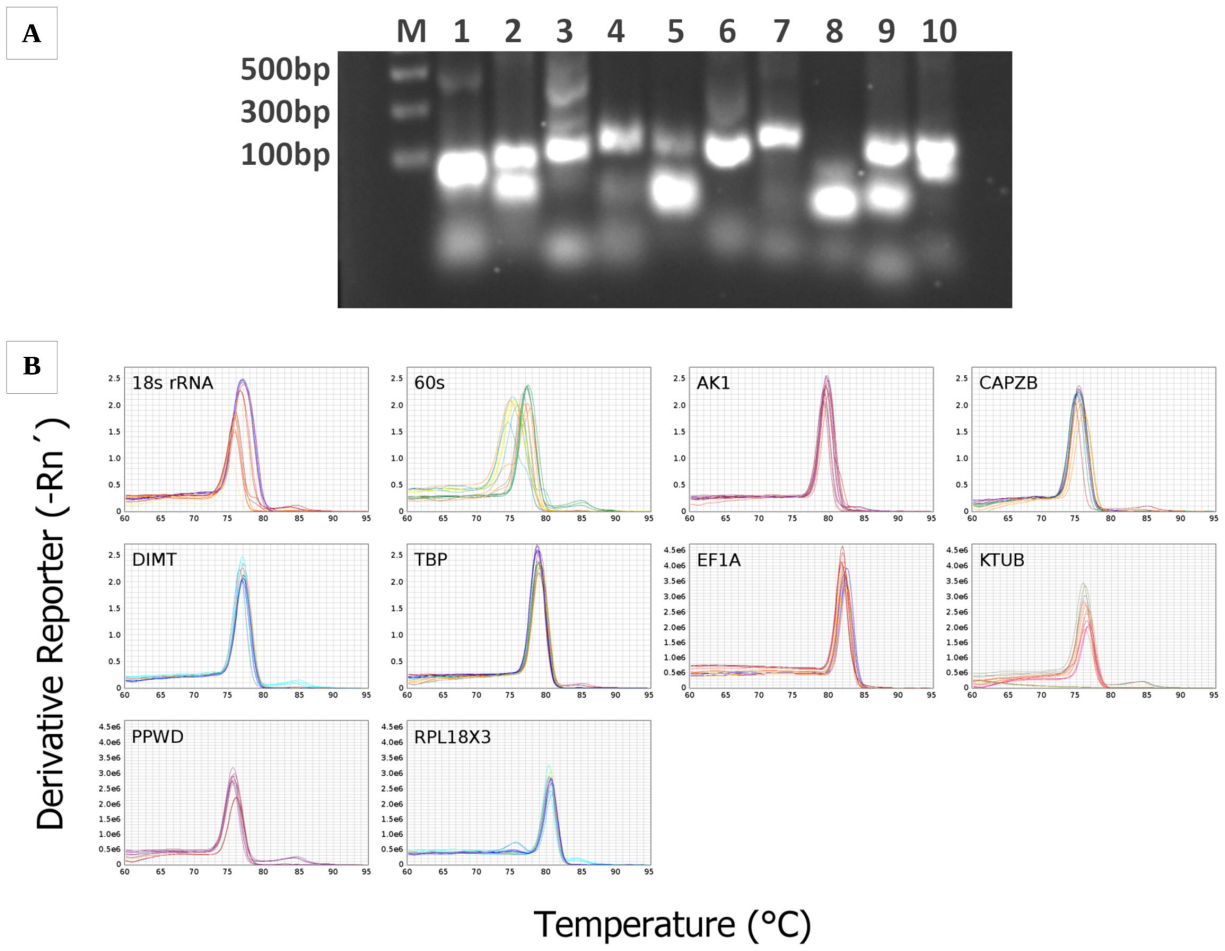
Confirmation of amplicon size and primer specificity. **(A)** Amplification results for *Vespula vulgaris* candidate reference genes from cDNA template. Columns 1–10 respectively represent *18s rRNA*, *60s*, *AK1*, *CAPZB*, *DIMT*, *TBP*, *EF1A*, *KTUB*, *PPWD*, *RPL18X3*, and M is the DNA ladder. **(B)** Melt curve analysis of all *Vespula vulgaris* candidate reference genes. All RT-qPCR products had a single melting curve suggesting the breakdown of only one PCR product.

The primer specificity was validated by using 1% agarose gels (**Figure 1A**). Primer specificity of all candidate reference genes was further tested via the amplification and dissociation curve analysis of every gene, visualized with a single peak and no detectable signal in the negative control (**Figure 1B**).

Standard curves were generated for every gene based on a five-fold serial dilution of the pooled cDNA (**Figure 1B**). The correlation coefficient (R2) values ranged from 0.8–1, and the PCR efficiency values determined by the standard curve ranged from 81.41%–268.42% (**Table 1**). From this data, the six following genes were selected for further analyses: *CAPZB, KTUB, RPL18X3, AK1, EF1A*, and *TBP*.

### 3.2 Expression profiling of candidate reference genes

**Figure 2:**
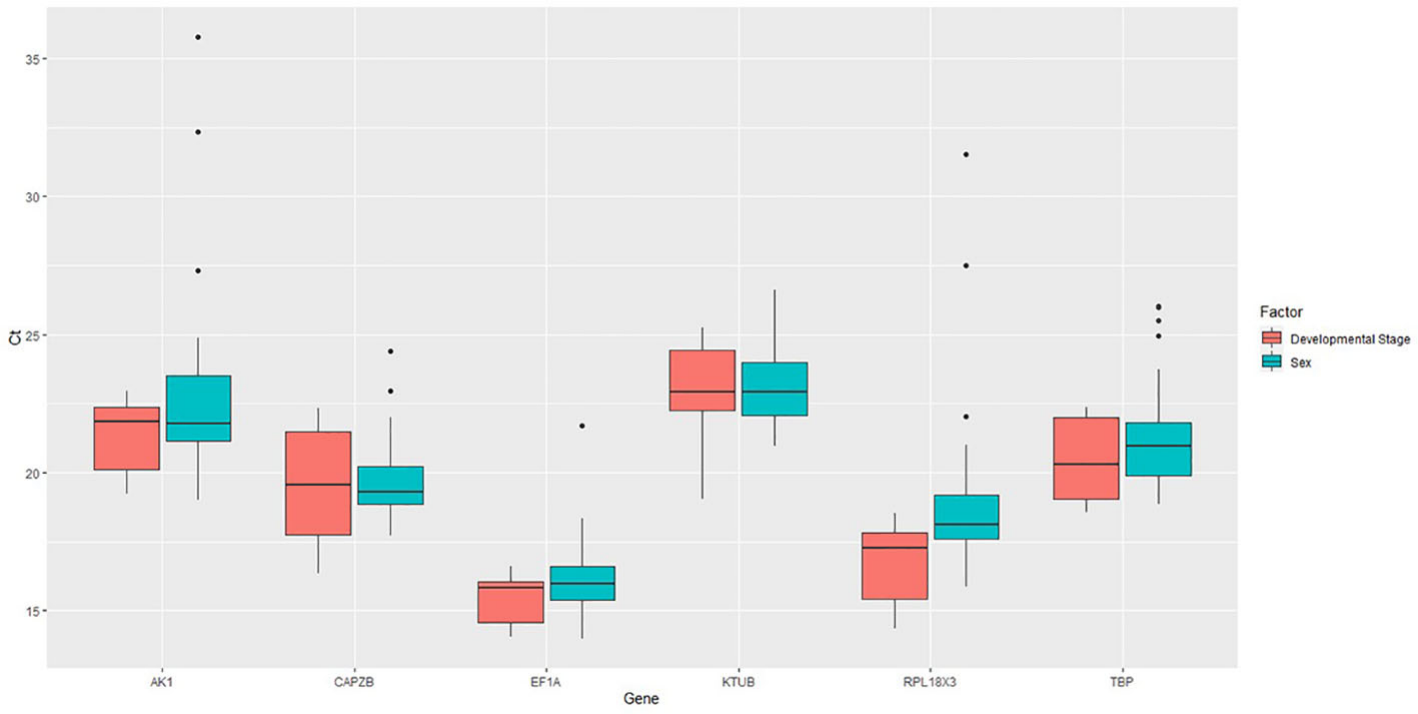
Expression profiles of candidate reference genes in both treatments. Box plot analysis showing the raw Ct values of candidate reference genes in *V. vulgaris*.

Expression levels were defined as the number of amplification cycles required to reach a fixed threshold in the exponential phase of the PCR reaction (26). Thresholds were automatically set by the instrument software in all genes to determine the Ct values. These expression levels were tested across two treatments in *V. vulgaris*, with variable Ct values highlighting a range of expression levels and varied expression patterns for these six reference genes (**Figure 2**).

The expression levels of *CAPZB, RPL18X3*, and *TBP* showed greater variability within the developmental stage, while the Ct values for *AK, KTUB*, and *TBP* were the most varied in the sexes. *EF1A* and *KTUB* showed the narrowest range of variability between factors, suggesting a relatively stable expression at developmental stages and sexes (**Figure 2**).

### 3.3 Expression stability and ranking of candidate genes

All candidate reference genes were ranked based on the stability of gene expression for both treatments via four statistical algorithms.

*BestKeeper* ranked the candidate reference genes *AK1* as the most stably expressed candidate reference gene in both developmental stage and sex, and *CAPZB* as the least stably expressed candidate reference gene in both conditions. Based on the *ΔCt* method ranking system, the candidate reference genes *TBP* and *CAPZB* were the most stably expressed genes for developmental stage and sex, respectively. Analysis of candidate reference genes ranked *EF1A* as the most stable gene based on both developmental stage and sex expression profiling. Using the analysis tool *NormFinder*, genes *TBP* and *KTUB* were ranked as the most stable genes across developmental stages and sexes, respectively. The overall values of the stability of the six selected candidate reference genes can be viewed in **Table 2**.

**Table 2:** Stability of the top six reference genes from four different variables.

Experimental conditions	Reference gene	*BestKeeper*		*ΔCt*		*NormFinder*		*geNorm*		*RefFinder*	
		Stability	Rank	Stability	Rank	Stability	Rank	Stability	Rank	Stability	Rank
Sexes	*AK1*	0.29	1	3.13	4	0.82	6	2.8	6	4.90	5
	*CAPZB*	0.78	6	2.44	1	0.34	2	1.41	2	4.16	4
	*EF1A*	0.70	5	3.36	6	0.43	3	1.33	1	1.41	2
	*KTUB*	0.65	4	2.67	3	0.30	1	1.44	3	5.18	6
	*RPL18X3*	0.60	2	3.21	5	0.68	5	2.28	5	3.41	3
	*TBP*	0.64	3	2.62	2	0.51	4	1.69	4	1.19	1
Developmental stage	*AK1*	0.63	1	3.29	2	0.69	6	1.24	6	6.00	6
	*CAPZB*	0.98	6	2.38	3	0.52	5	1.06	4	2.45	3
	*EF1A*	0.89	3	2.80	5	0.49	4	0.75	1	2.11	2
	*KTUB*	0.91	5	2.64	6	0.45	2	1.12	5	1.73	1
	*RPL18X3*	0.89	2	3.17	4	0.45	3	0.77	2	3.98	5
	*TBP*	0.91	4	2.62	1	0.35	1	0.83	3	2.83	4

### 3.4 Comprehensive ranking of selected reference genes via *RefFinder*

After the expression stabilities for developmental stage and sex were analysed by the above four methods, *RefFinder* was employed to calculate an overall stability ranking and therefore the most appropriate reference genes for these experimental conditions.

**Figure 3:**
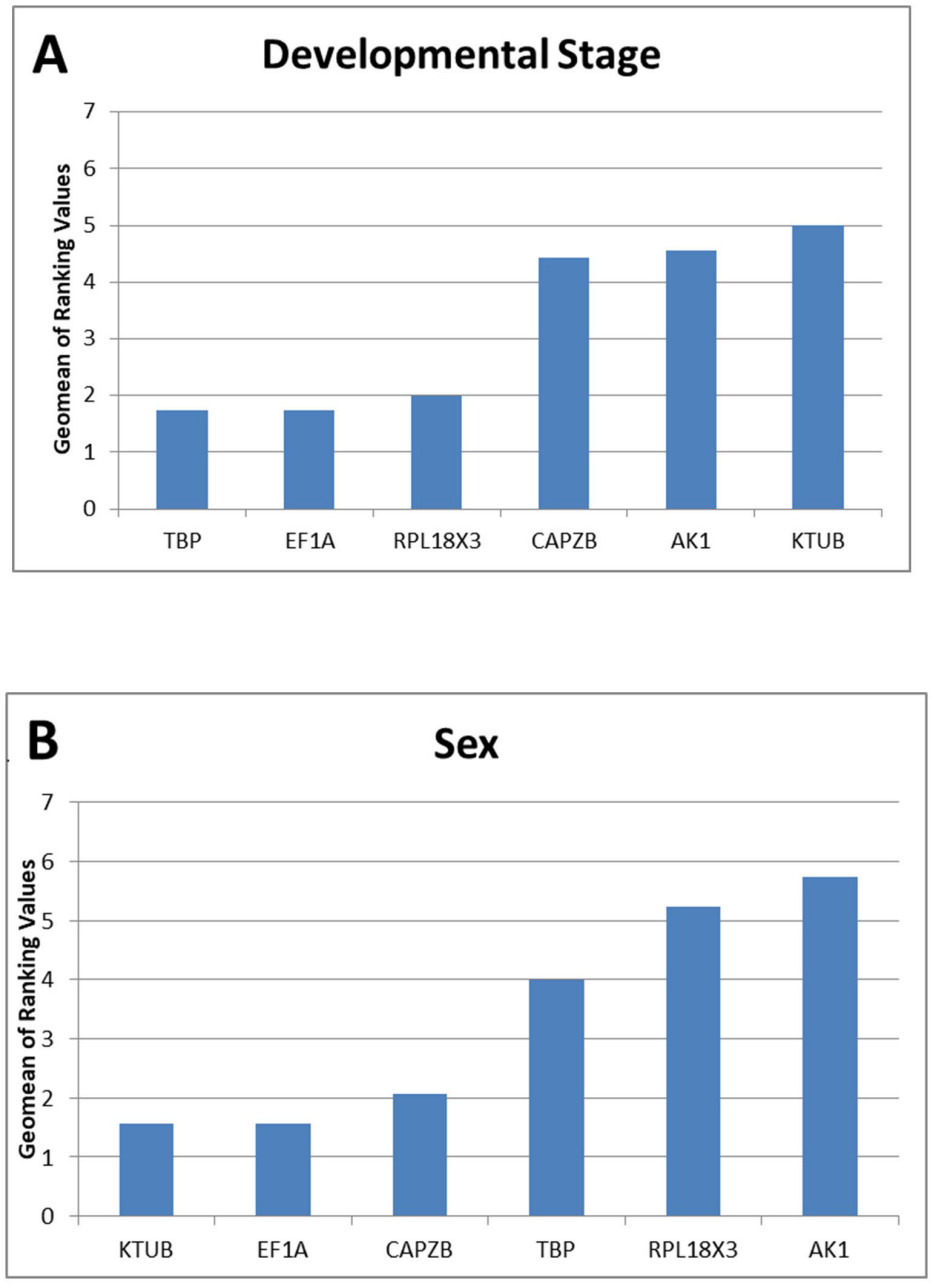
Stability of candidate reference gene expression under developmental stage **(A)** and sex **(B)** treatments. Lower Geomean values indicate more stable expression based on *RefFinder* analyses.

For developmental stages, analyses using the Δ*Ct* method and *NormFinder* both ranked the candidate reference gene *TBP* as the most stable, while *BestKeeper* and *geNorm* ranked *AK1* and *EF1A* as the most stable genes, respectively. In sex, the most stable gene varied for each form of analysis, with *AK1* for *BestKeeper*, *CAPZB* for the Δ*Ct* method, *KTUB* when ranked by *NormFinder*, and *EF1A* when using *geNorm* (**Table 1**). These algorithms varied in the ranking of the most unstable genes, putting *AK1* as the least stable in sex for all measurements bar *BestKeeper*, while *NormFinder* and *geNorm* consistently ranked *AK1* as the least stable gene under the sex condition. *RefFinder* ranked the stability of these six genes from most to least stable in the developmental stage *TBP>EF1A>RPL18X3>CAPZB> AK1>KTUB* (**Figure 3A**) and as *KTUB>EF1A>CAPZB>TBP>RPL18X3>AK1* under the treatment for sex (**Figure 3B**). *geNorm* attempted to determine an optimal number of reference genes required to normalise target gene data, but could not recommend an amount as the variability between sequential normalisation factors (based on the n and n+1 least variable reference targets) is relatively high (*geNorm* V >0.15). Additional candidate reference genes were recommended, or if not possible, to use the four reference targets with the lowest M value when running an experiment under the developmental stage condition, or three reference targets with the lowest M value for experiments concerning sex. This was recommended as using multiple (in this case, non-optimal) reference targets would result in more accurate normalization compared to using a single nonvalidated reference gene.

### 3.5 Validation of selected reference genes in *V. vulgaris*

**Figure 4:**
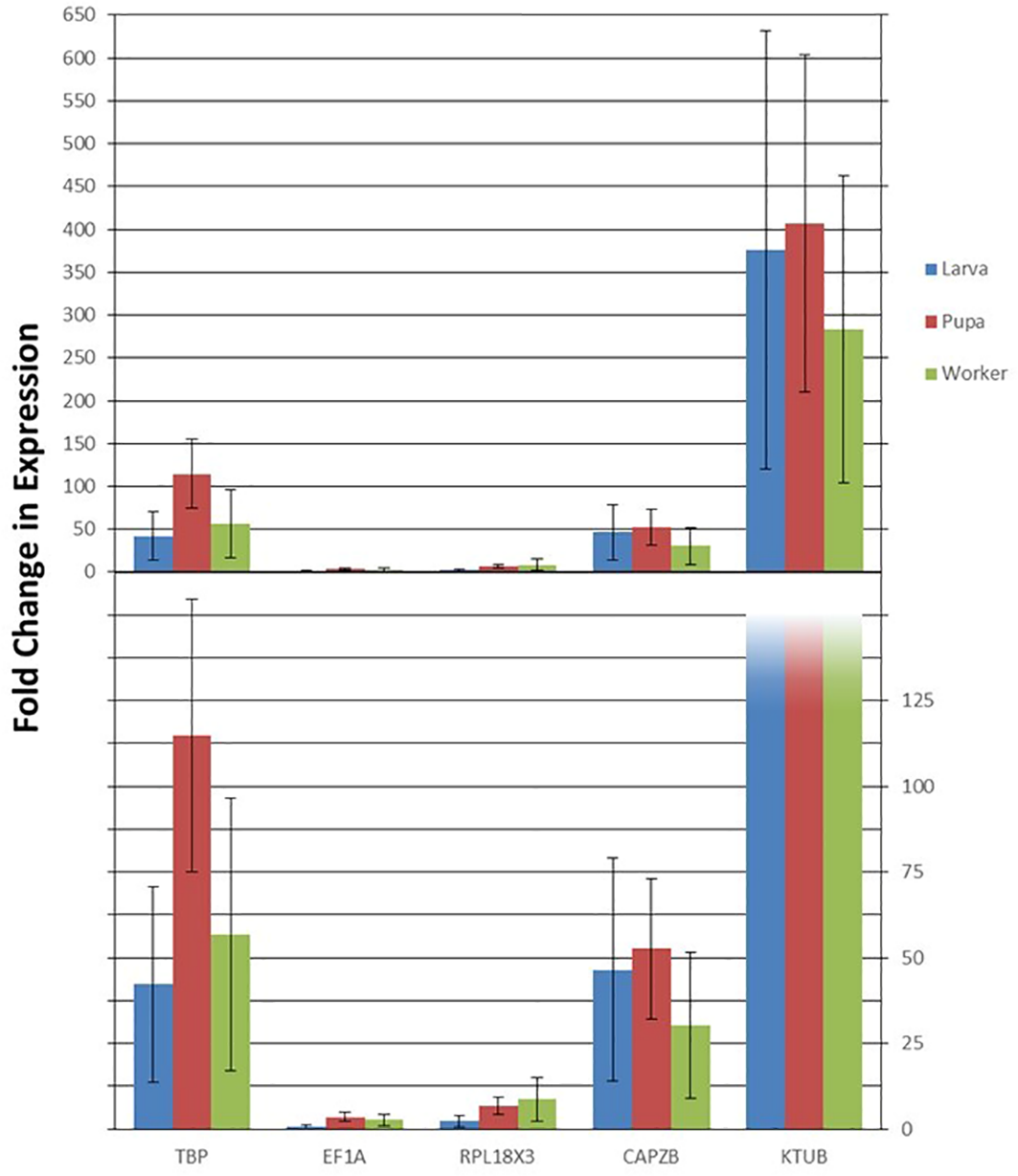
Validation of the candidate reference genes. Relative expression levels of the target gene RPB7 in different samples using different normalization factors (the four most and one least stable genes). Error bars represent the means and standard deviation of three biological replicates.

The findings from this study show a striking difference in the stability of candidate reference genes from one condition to the other in *V. vulgaris*. While a comprehensive ranking by *RefFinder* showed that *KTUB* expressed the highest stability between sexes, it also indicated that the same gene had the most varied stability under the developmental stages condition. *TBP* and *EF1A* were considered the most stably expressed gene for the developmental stages condition. The expression patterns of *RPB7* across various life stages were inconsistent when normalized with these two most stable reference genes (**Figure 4**). Transcripts of *RPB7* Ct values showed some variation across all developmental stages, while normalization of transcripts using *TBP* and *CAPZB* highlighted peak *RPB7* expression in pupae (**Figure 4**). Overall, *RPB7* had varied expression patterns, with over 400-fold more expression when normalized with the least stable *KTUB* gene, compared to a less than four-fold change in expression when normalized with *EF1A* (**Figure 4**).

## 4 Discussion

To further investigate the biological function of a gene in an organism, quantification of that gene is crucial and requires experiments run in conjunction with reference genes of high stability. There have been several recent publications on selecting suitable reference genes for Hymenoptera (27–32), all highlighting the variability of such reference genes across a suite of experimental conditions. This paper aimed to find suitable reference genes for RNAi experiments in *V. vulgaris*, using five different algorithms to identify the stability of six reference genes. To date, there has been no investigation into appropriate reference genes for gene expression research in this social wasp. The genes selected for this study are regularly used as reference genes in other insect gene expression papers and were thus selected for this study. I chose six reference genes as candidate genes to examine their stability under different developmental stages and sexes. In an effort to reduce errors in analysis caused by selecting co-regulated transcripts, the candidate genes were ranked according to four statistical models (*BestKeeper*, *ΔCt* method, *geNorm*, and *NormFinder*).

The comparative analyses showed some consistency across algorithms under both experimental conditions. *NormFinder* and *geNorm* produced highly similar results, while *BestKeeper* had the greatest discrepancies in comparison to the other algorithms; this finding was consistent with those of Gao et al. (17). Such fluctuations in ranking orders from these programs can make it difficult for researchers to select the optimal reference genes. Therefore, I used *RefFinder* for a final ranking, as this algorithm incorporates all of the above-mentioned algorithms for a final overall rank of candidate reference genes.

The final analysis conducted by *RefFinder* was used to rank the overall stability of the selected candidate reference genes and demonstrated that *TBP, EF1A, RPL18X3*, and *CAPZB* were the most stable genes under the developmental stage treatment, and *KTB*, *EF1A*, and *CAPZB* exhibited the highest stability values among sexes.

The findings here emphasize the instability of such reference genes in different conditions, and that we should not expect to have perfect reference genes across all conditions, and further reinforce that there should never be a single universal reference gene employed for expression analysis across all experimental conditions and species. It should be said that this does not mean that reference genes are pointless, but that they need to be explored so that the right ones can be implemented for appropriate experiments. In my study, *EF1A* expressed the most stability in both developmental stage and sex conditions, consistent with findings of reference gene analysis in the Sugarcane Stem Borer *Chilo sacchariphagus* (33), *Tamarixia radiate* (31) and *Glenea cantor* (34). This study showed that the expression of *TBP* was highly stable across developmental stages, but should not be used in studies involved with different sexes. *KTUB* had the most extreme levels of expression, as it was the least stable gene across developmental stage experiments, but then the most stable when tested under the condition of sex. *CAPZB* remained in the middle range for both conditions, while *RPL18X3* flanked the upper and lower levels of stability in developmental stage and sex, respectively. *AK1* was a consistently poor performer and should not be used for either condition.

One limitation of the algorithms used is that *NormFinder, geNorm*, and *RefFinder* cannot conduct analyses with missing data points, thus whole samples have to be discarded (16, 22, 24). *BestKeeper* and the *ΔCt* method do not have this issue, however, *BestKeeper* is limited in that it can only analyze a maximum of ten genes (21). Ideally, I would have liked to include additional conditions to test the reference genes on, such as tissues, temperature, and diet. However, limited samples, time constraints, and reagent costs made this unfeasible. Future work would greatly benefit from including these parameters in their analyses.

The gene *RPB7* is a catalyst for transcription of DNA into RNA using four ribonucleoside triphosphates as substrates. The protein comprises of mobile elements that synthesis mRNA precursors and bind single-stranded DNA and RNA (35). The four most stable and one least stable reference gene underwent validation by determining the expression profiles of the target gene, RPB7. The expression level of RPB7 after normalization drastically varied across all five of these genes, suggesting they could not honestly reflect its expression level (**Figure 4**). These results are inconsistent with the gene normalizer concept, implying that using these reference genes will not normalize gene expression data in *V. vulgaris.*

Overall, this qPCR study of suitable reference genes for expression research in *V. vulgaris* has provided the first building blocks necessary. A lack of stability has been found, rendering many of these candidate reference genes unsuitable. Potentially, it may not be possible to identify suitable reference genes for qPCR analysis, thus the two most stable reference genes under the condition developmental stage would be preferable to no species-specific reference at all. More research into additional reference genes, in combination with more treatment conditions such as tissues and diet, would not go amiss for additional insights.

## Data Availability

The datasets presented in this study can be found in online repositories. The names of the repository/repositories and accession number(s) can be found in the article/supplementary material.
